# *Drosophila melanogaster* Toll-9 elicits antiviral immunity against Drosophila C virus

**DOI:** 10.1128/jvi.02214-24

**Published:** 2025-05-14

**Authors:** Manish Chauhan, Peter E. Martinak, Benjamin M. Hollenberg, Alan G. Goodman

**Affiliations:** 1 School of Molecular Biosciences, College of Veterinary Medicine, Washington State University 196198, Pullman, Washington, USA; 2 Elson S. Floyd College of Medicine, Washington State University 445950, Spokane, Washington, USA; 3 Paul G. Allen School for Global Health, College of Veterinary Medicine, Washington State University 663014, Pullman, Washington, USA; Wageningen University & Research, Wageningen, the Netherlands

**Keywords:** DCV, AKT, endosome, dsRNA, *Dicer2*, JAK/STAT

## Abstract

The Toll pathway plays a pivotal role in innate immune responses against pathogens. The evolutionarily conserved pattern recognition receptors (PRRs), including Toll-like receptors (TLRs), play a crucial role in recognition of pathogen-associated molecular patterns (PAMPs). The *Drosophila* genome encodes nine Toll receptors that are orthologous to mammalian TLRs. While mammalian TLRs directly recognize PAMPs, most *Drosophila* Tolls recognize the proteolytically cleaved ligand Spätzle to activate downstream signaling cascades. In this study, we demonstrated that Toll-9 is crucial for antiviral immunity against Drosophila C virus (DCV), a natural pathogen of *Drosophila*. A transposable element insertion in the *Toll-9* gene renders the flies more susceptible to DCV. The stable expression of Toll-9 in *Drosophila* S2 cells results in increased *Dicer2* induction and reduced AKT phosphorylation, collectively establishing an antiviral state that inhibits DCV replication. Toll-9 localizes to endosomes, where it binds viral double-stranded RNA (dsRNA), highlighting its role in detecting viral replication intermediates. Together, these findings identify Toll-9 as a key player in antiviral immunity against DCV infection, acting through its ability to recognize dsRNA and drive *Dicer2* expression, along with other AKT-mediated antiviral responses.

**IMPORTANCE:**

Insects rely on innate immunity and RNA interference (RNAi) to combat viral infections. Our study underscores the pivotal role of *Drosophila* Toll-9 in antiviral immunity, aligning with findings in *Bombyx mori*, where Toll-9 activation upregulates the RNAi component *Dicer2*. We demonstrate that *Drosophila* Toll-9 functions as a pattern recognition receptor (PRR) for double-stranded RNA (dsRNA) during Drosophila C virus (DCV) infection, akin to mammalian Toll-like receptors (TLRs). Toll-9 activation during DCV infection leads to the upregulation of *Dicer2* and *Argonaute2* and dephosphorylation of AKT. This study also reveals that Toll-9 localizes in endosomal compartments where it interacts with dsRNA. These insights enhance our understanding of *Drosophila* innate immune mechanisms, reflecting the evolutionary conservation of immune responses across diverse species and providing impetus for further research into the conserved roles of TLRs across the animal kingdom.

## INTRODUCTION

The innate immune system is the first line of defense against invading pathogens, and since insects lack adaptive immunity, they rely solely on innate immunity (1). The innate immune system is an efficient and fast-acting defense mechanism that identifies pathogen-associated molecular patterns (PAMPs) through pattern recognition receptors (PRRs) (2, 3). Upon interacting with PAMPs, PRRs initiate downstream signaling to mount an antimicrobial response (4, 5). In higher vertebrates such as mammals, Toll-like receptors (TLRs) recognize PAMPs such as lipopolysaccharide (LPS), fungal zymosan, and cytosolic nucleic acids (6–8).

The Toll receptor was first discovered as a type I transmembrane receptor for its role in dorsoventral patterning during embryonic development in *Drosophila*. Later, its role in innate immunity was deciphered (2, 9). Toll-1 was identified as an important immune receptor for its role in activating antimicrobial peptides (AMPs) against Gram-positive bacteria and fungi. There are nine *Toll* genes in *Drosophila*, and *Toll-1* has a major role in innate immune signaling (10, 11). Furthermore, studies suggest Toll-8 (Tollo) mediates immunity in the trachea of *Drosophila*, and Toll-7 regulates anti-viral responses independent of an NF-κB signaling pathway (12, 13). However, the immunological function of the remaining *Drosophila* Toll receptors has not yet been deciphered (14, 15).

Of the nine *Drosophila* Tolls, Toll-9 is most closely related to mammalian TLRs and is orthologous to TLR10 (14). Toll-9 lacks multiple autoinhibitory cysteine-rich motifs, which differentiates it from other Toll receptors and makes it more similar to mammalian TLRs (16, 17). Previous studies on Toll-9 suggest it has a role in innate immunity, and Ooi et al. have shown that overexpression of Toll-9 induces the production of AMPs (18). However, Narbonne-Reveau et al. demonstrated that in the absence of Toll-9, there was no change in basal induction levels of AMPs. In addition, Toll-9 overexpression was unable to induce AMP production during oral bacterial infection (19). This finding was contrary to the study by Ooi et al., which showed that Toll-9 induces basal levels of AMPs and that its overexpression further increases AMP production. Recent studies have highlighted the role of Toll-9 in cell fitness. Specifically, the activation of Toll-9 signaling has been shown to induce apoptosis during cell competition in developing tissues, thereby eliminating unfit cells and promoting proliferation in surviving cells (20, 21). Furthermore, Toll-9 has been shown to regulate aging and neurodegenerative processes in a *Drosophila* model of tauopathy (20, 22).

Arthropods rely on the recognition of virus-derived double-stranded (dsRNA) to activate the RNA interference (RNAi) pathway and mount antiviral responses (23, 24). Drosophila C virus (DCV) is a positive-sense RNA virus of the family Dicistroviridae and is one of the most well-described *Drosophila* viruses. Since *Drosophila*’s major antiviral pathway is mediated through RNAi, and DCV generates dsRNA replication intermediates that are detected by RNAi components such as *Dicer2* to cleave the viral dsRNA (25–27), we hypothesized that *Drosophila* encodes a PRR that binds to dsRNA during DCV infection. A previous study indicates that TLR10 recognizes dsRNA in the endosome and competes for dsRNA binding with TLR3 (28). Toll-9 is orthologous to TLR10, so we further hypothesized that Toll-9 is a PRR for dsRNA during DCV infection. As such, we found that a transposable element insertion in *Toll-9* renders flies more susceptible to DCV infection compared to isogenic control flies. We overexpressed Toll-9 in S2 cells and found that it results in reduced DCV replication that is correlated with increased *Dicer2* expression levels. We observed that Toll-9 colocalizes with Rab5 (early endosome) and Rab7 (late endosome) in the endosome and binds to dsRNA. We also observed that Toll-9 expression during DCV infection leads to a reduction in AKT phosphorylation. Altogether, our data suggest Toll-9 plays a crucial role in antiviral immunity upon binding dsRNA that leads to reduced AKT phosphorylation and increased induction of *Dicer2*.

## RESULTS

### Toll-9 mutant flies display increased susceptibility to DCV infection

**Fig 1:**
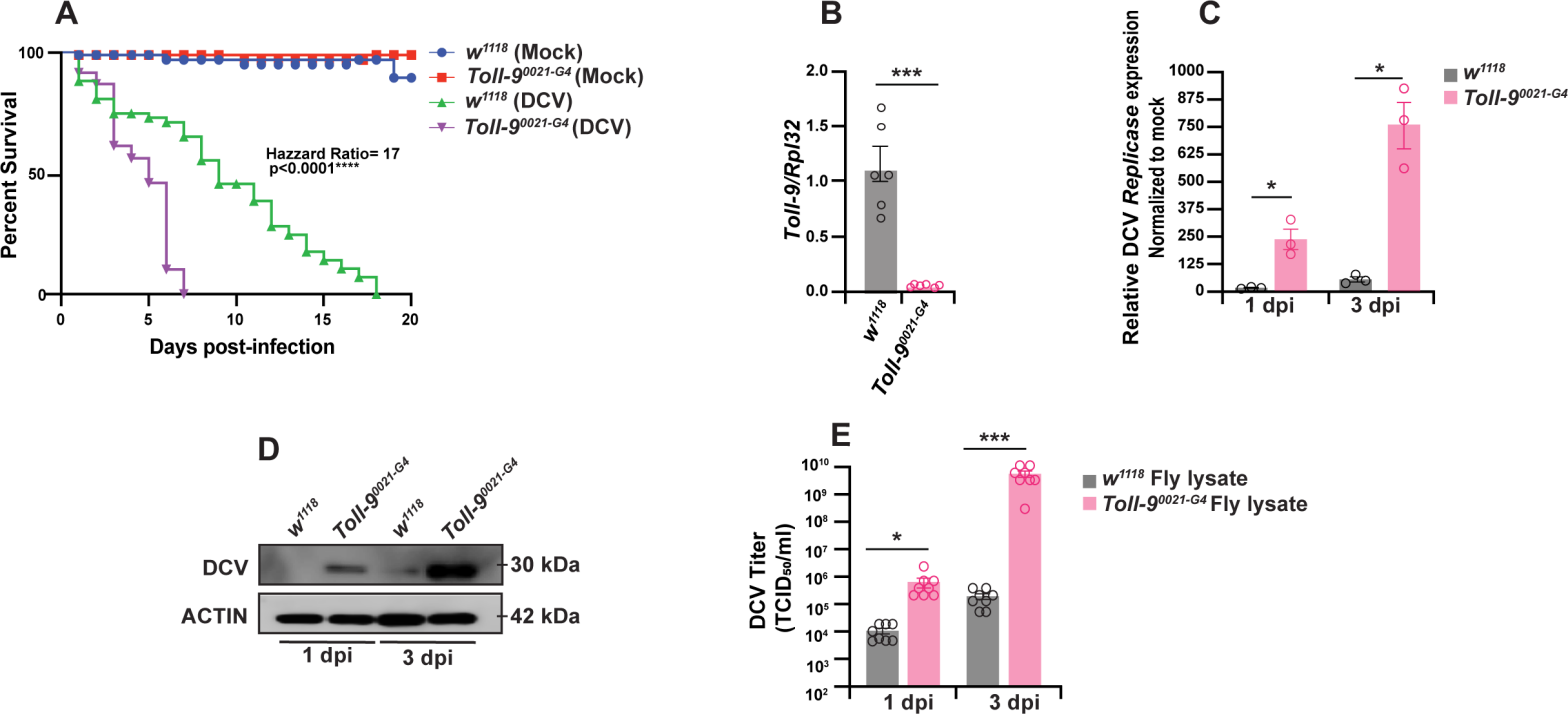
Toll-9 mutant flies display increased susceptibility to DCV infection. (**A**) Survival analysis for isogenic control *w^1118^* flies and *Toll-9^0024-G4^* mutant flies injected with phosphate-buffered saline (PBS) and DCV (*N* = 80 flies from three independent experiments). (**B**) qRT-PCR analysis of *Toll-9* expression in *w^1118^* flies and *Toll-9^0024-G4^* mutant flies. Data are representative of six biological replicates (groups of 3–5 flies) from three independent experiments. Error bars, SEM. Unpaired t test, ***P < 0.001. (**C**) qRT-PCR analysis of DCV-infected *w^1118^* flies and *Toll-9^0024-G4^* mutant flies at 1 and 3 dpi for DCV mRNA. Data are representative of three biological replicates (groups of 3–5 flies), each from three independent experiments. Error bars, SEM. One-way analysis of variance (ANOVA), *P < 0.05. (**D**) Western blot analysis of DCV capsid protein with anti-DCV antibody from fly lysate (groups of 3–5 flies) of isogenic control *w^1118^* flies and *Toll-9^0024-G4^* mutant flies injected with DCV at 1 and 3 dpi. Blots are representative of three independent experiments. (**E**) DCV titer in S2 cells infected with fly lysate from isogenic *w^1118^* flies and *Toll-9^0024-G4^* mutant flies injected with DCV calculated as 50% tissue culture infectious dose (TCID_50_/mL). Data are representative of eight biological replicates (groups of 3–5 flies) from four independent experiments. Error bars, SEM. One-way ANOVA, *P < 0.05; ***P < 0.001.

We investigated the role of Toll-9 in *Drosophila* antiviral defense by infecting isogenic control *w^1118^* and transposable element insertion mutant *Toll-9^0024-G4^* flies with DCV. We observed that transposable element insertion mutant *Toll-9^0024-G4^* flies are more susceptible to DCV infection because they exhibit higher mortality rates compared to *w^1118^* isogenic control flies (Fig. 1A). To validate the reduced expression of *Toll-9* in the transposable element insertion mutant *Toll-9^0024-G4^* flies, we performed quantitative reverse transcription PCR (qRT-PCR) analysis. Our results confirmed a significant downregulation of *Toll-9* expression in *Toll-9^0024-G4^* mutant flies compared to isogenic control *w^1118^* flies (Fig. 1B). Next, we examined DCV replication in *Toll-9^0024-G4^* mutant flies compared to control *w^1118^* flies by measuring intracellular DCV RNA and capsid protein levels. Our analysis revealed that the levels of DCV *replicase ORF1* mRNA were significantly higher in *Toll-9^0024-G4^* mutant flies at both 1- and 3-days post-infection (dpi) compared to the control *w^1118^* flies (Fig. 1C). We also observed elevated levels of the DCV capsid protein in *Toll-9^0024-G4^* mutant flies infected with DCV at both 1 and 3 dpi, further indicating increased viral replication in the absence of functional Toll-9 (Fig. 1D). Additionally, we quantified the levels of infectious virus in DCV-infected control *w^1118^* flies and *Toll-9^0024-G4^* mutant flies. Our results indicate that DCV titers were higher in *Toll-9^0024-^*^G*4*^ mutant flies than *w^1118^* flies at both 1 and 3 dpi (Fig. 1E). Together, these results indicate that *Toll-9* is involved in antiviral immunity against DCV infection in *Drosophila,* and mutation in *Toll-9* renders flies more susceptible to DCV infection and exhibits increased viral load.

### *Toll-9* regulates the expression of *Dicer2* and *Argonaute2*

**Fig 2:**
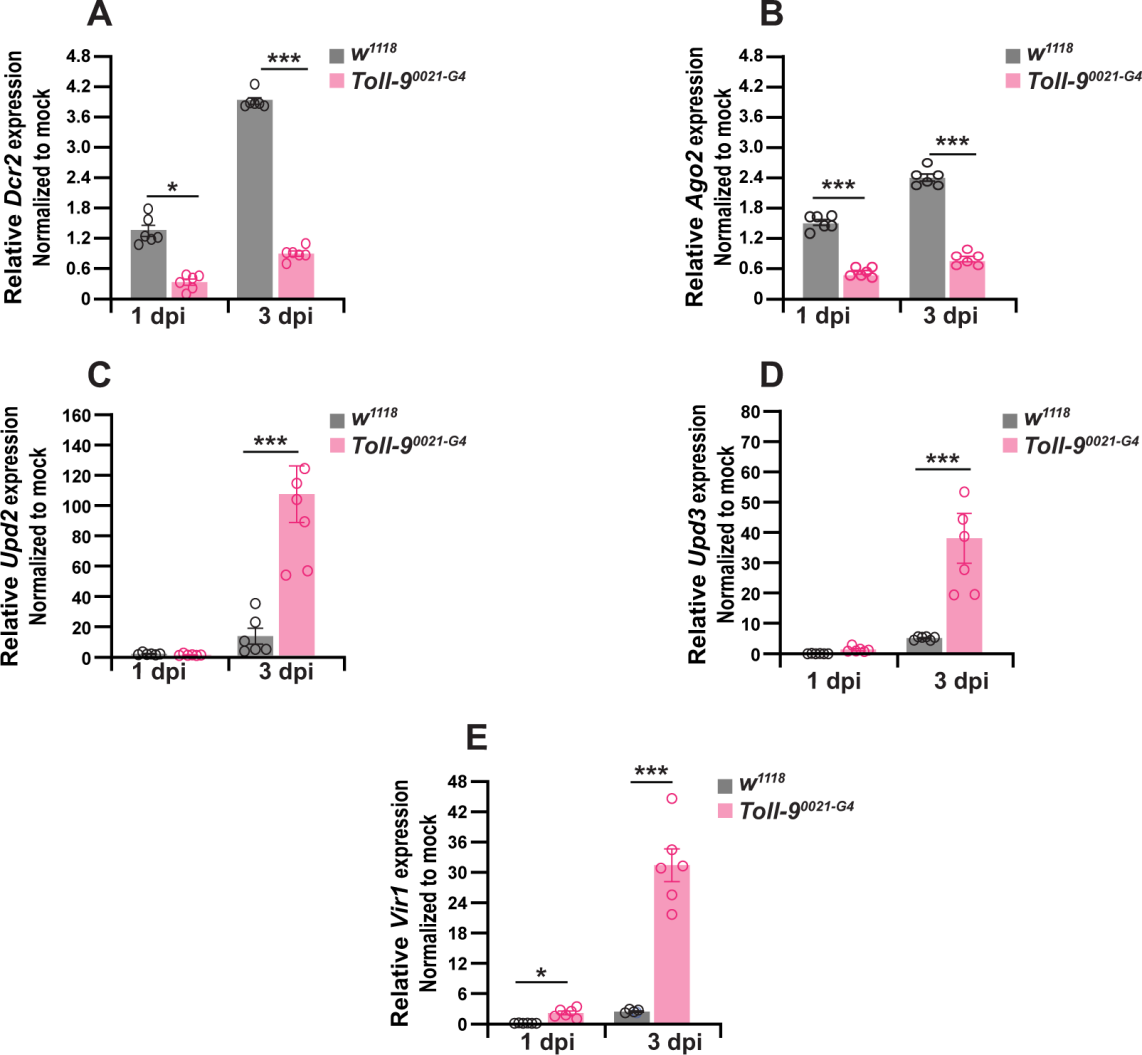
*Toll-9* regulates the expression of RNAi and JAK/STAT genes. qRT-PCR analysis of DCV-infected *w^1118^* flies and *Toll-9^0024-G4^* mutant flies at 1 and 3 dpi for (**A**) *Dicer2*, (**B**) *Argonaute2*, (**C**) *Upd2*, (**D**) *Upd3*, and (**E**) *Vir1*. For the mock infection, both *w^1118^* and *Toll-9^0024-G4^* mutant flies were injected with PBS and collected at indicated time points for normalization. Data are representative of six biological replicates (groups of 3–5 flies) from three independent experiments. Error bars, SEM. One-way ANOVA, *P < 0.05; ***P < 0.001.

To address the role of Toll-9 in antiviral immunity and the control of DCV infection *in vivo*, we examined host gene expression in DCV-infected *w^1118^* isogenic control and *Toll-9^0024-G4^* mutant flies by qRT-PCR. A previous study demonstrated that *Drosophila* Dicer2 plays a pivotal role in antiviral defense against RNA viruses from three distinct families, including DCV. Notably, loss-of-function mutations in *Dicer2* significantly increase the susceptibility of flies to viral infections (29). We observed that DCV-infected *Toll-9^0024-G4^* mutant flies have lower expression of genes *Dcr2* and *Ago2* at both 1 and 3 dpi compared to *w^1118^* isogenic control flies (Fig. 2A and B). The JAK/STAT pathway also provides antiviral defense in *Drosophila* (30). During viral infection, the JAK/STAT pathway is activated when the upd2 and upd3 ligands bind to the Domeless receptor to activate STAT92E and induce expression of *Vir1* (31, 32). During DCV infection, *Toll-9^0024-G4^* mutant flies exhibited an elevated expression of JAK/STAT pathway-related genes compared to *w^1118^* control flies on both 1 and 3 dpi (Fig. 2C through E). It was previously shown that while the JAK/STAT pathway is an indicator of DCV infection, it was not sufficient for triggering an antiviral response to DCV (33). Similarly, our results show that the upregulation of JAK/STAT signaling in DCV-infected *Toll-9^0024-G4^* mutant flies is insufficient to produce the antiviral state observed in wild-type flies, which is primarily driven by the induction of *Dicer2* and *Argonaute2*. The inability of JAK/STAT signaling to compensate for the loss of RNAi gene induction may drive the enhanced DCV replication and increased susceptibility to infection observed in the *Toll-9^0024-G4^* mutant flies.

### Stable expression of *Toll-9* reduces DCV infection in *Drosophila* S2 cells

**Fig 3:**
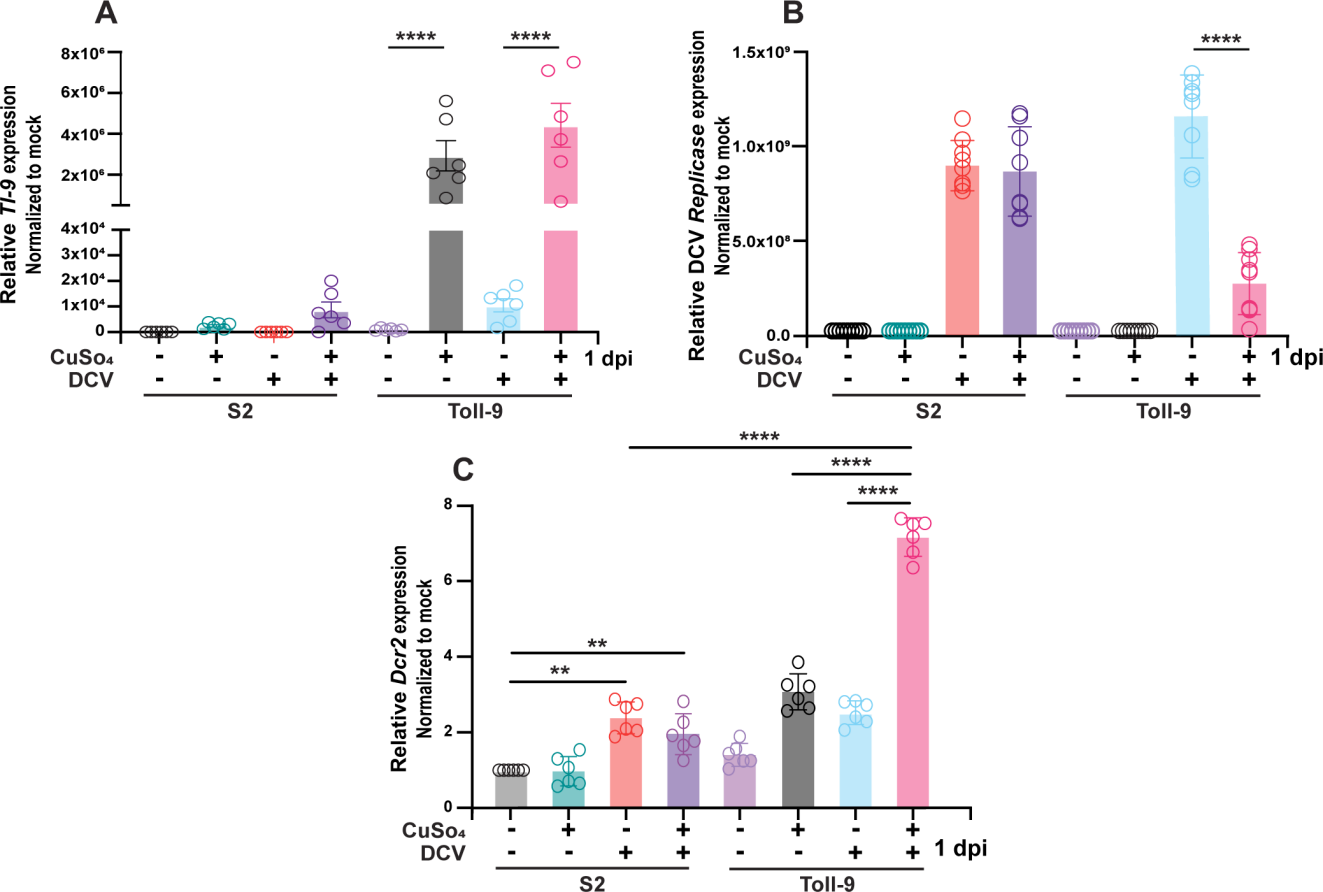
Stable expression of *Toll-9* reduces DCV infection in *Drosophila* S2 cells. qRT-PCR analysis of DCV-infected naïve S2 and Toll-9 OE cells in the presence and absence of CuSO_4_ at 1 dpi to detect expression of (**A**) *Toll-9*, (**B**) DCV *replicase ORF 1*, and (**C**) *Dicer2* mRNA. Data are representative of three biological replicates (well of cells) from three independent experiments. Error bars, SEM. One-way ANOVA, **P < 0.01; ****P < 0.0001.

To further elucidate the role of Toll-9 in defense against DCV infection, we generated a stable S2 cell line overexpressing Toll-9 under the control of a copper-inducible promoter. The construct included V5 and His tags to facilitate the verification of Toll-9 expression. To ensure optimal experimental conditions, clonal selection was performed to identify and isolate the clone with the highest Toll-9 expression upon copper induction while exhibiting minimal leaky expression in the absence of copper (Fig. 3A). The generation of a cell line stably expressing Toll-9 was necessary since modENCODE *Drosophila* cell line expression data deposited at FlyBase shows that *Toll-9* expression levels in S2 cells are low (34, 34–36). Upon DCV infection, S2 cells overexpressing Toll-9 (Toll-9 OE cells) displayed enhanced resistance compared to naïve S2 cells at 1 dpi, when induced with copper sulfate (CuSO_4_) (Fig. 3B). Previous studies established that *Dicer2* is a critical host susceptibility locus for viral infection, underscoring its essential role in *Drosophila’s* antiviral defense mechanism against DCV infection (29, 37). To investigate whether Toll-9’s antiviral effect is mediated through the induction of *Dicer2*, we assessed the expression of *Dcr2* in DCV-infected cells. We observed that CuSO_4_-induced Toll-9 OE cells exhibited significantly elevated expression of *Dcr2* compared to naïve S2 cells at 1 dpi (Fig. 3C). Overall, our findings suggest that Toll-9 plays a crucial role in antiviral defense during DCV infection of S2 cells by restricting viral replication through the induction of *Dicer2*.

### Modulation of AKT phosphorylation during DCV infection in the presence of Toll-9

**Fig 4:**
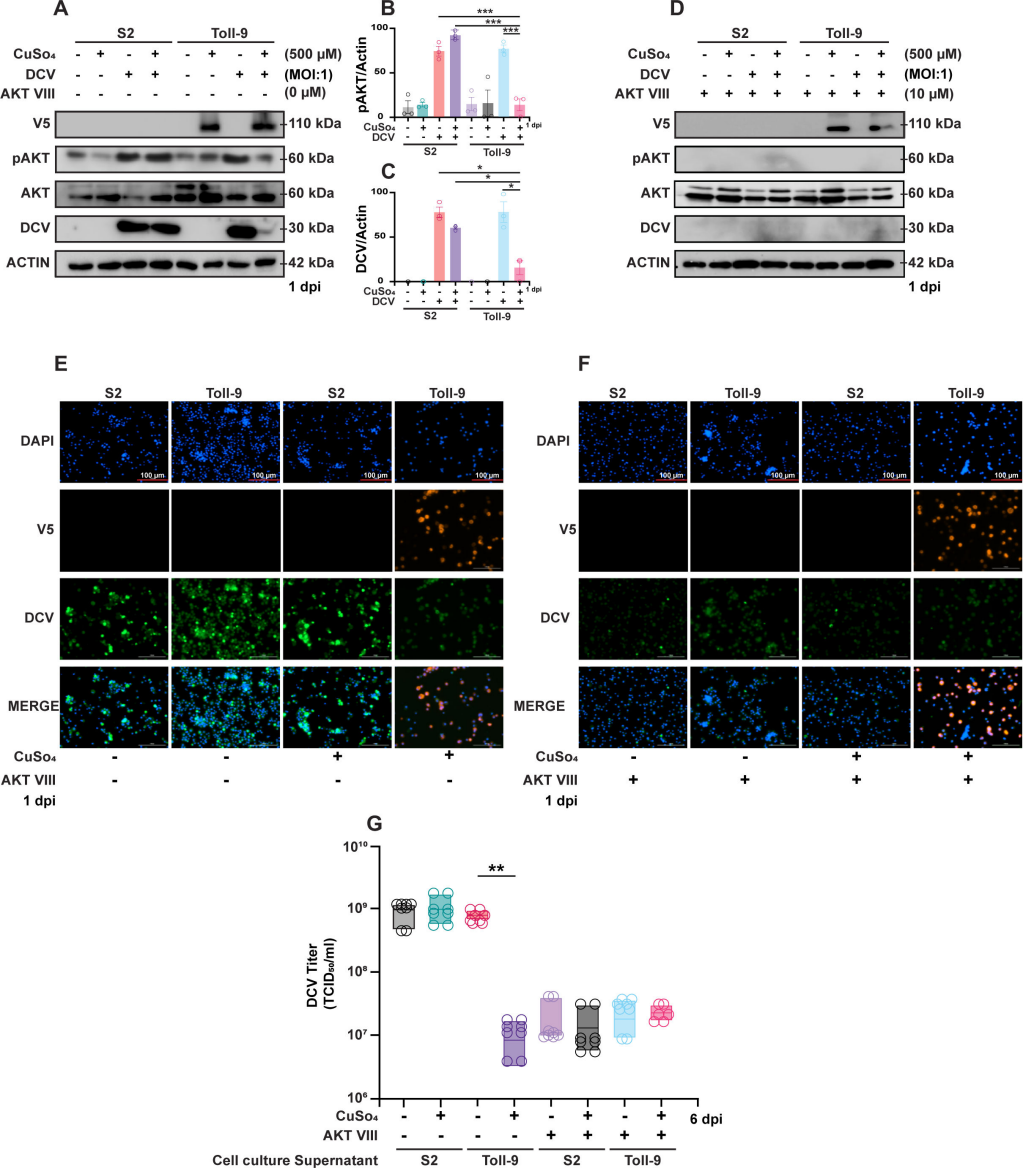
Toll-9 controls DCV infection via dephosphorylation of AKT. (**A**) Western blot analysis for indicated antibodies of cell lysate from DCV-infected S2 cells and Toll-9 OE cells in the presence and absence of CuSO_4_ at 1 dpi. Blots are representative of three independent experiments. (**B and C**) Densitometry quantitation of western blot showing expression for pAKT and DCV normalized to the expression of Actin. Data are representative of western blots from three independent experiments. Error bars, SEM. One-way ANOVA, *P < 0.05; ***P < 0.001. (**D**) Western blot analysis for indicated antibodies of cell lysate from DCV-infected S2 cells and Toll-9 OE cells in the presence and absence of CuSO4 at 1 dpi in the presence of AKT inhibitor (AKT VIII). Blots are representative of three independent experiments. (**E and F**) Micrographs showing the presence of DCV in naïve S2 cells and Toll-9 OE cells in the presence and absence of CuSO_4_ at 1 dpi in the presence and absence of AKT inhibitor. Data are representative of three independent experiments. (**G**) Viral titer of DCV-infected naïve S2 and Toll-9 OE cells in the presence and absence of AKT inhibitor calculated as TCID_50_/mL. Data are representative of eight biological replicates (well of cells) from four independent experiments. Error bars, SEM. One-way ANOVA, **P < 0.01.

AKT is a serine/threonine-specific protein kinase that plays a crucial role in multiple cellular processes: glucose metabolism, cell cycle progression, and protein synthesis (38). During viral infections, viruses hijack various cellular pathways to facilitate their own survival and replication. Numerous studies on different types of viruses, including DNA viruses, RNA viruses, and retroviruses, have demonstrated that these pathogens manipulate the PI3K/AKT pathway. This manipulation leads to the regulation of apoptosis, alteration of splicing mechanisms, modulation of endocytosis, enhancement of RNA synthesis, and reorganization of the actin cytoskeleton (39–44). A previous report on Sindbis virus (SINV) replication in *Drosophila* highlights the virus’s reliance on cellular AKT levels, emphasizing the critical role of AKT in the viral life cycle. Intriguingly, SINV infection has been shown to induce AKT phosphorylation. This increase in AKT phosphorylation may contribute to the facilitation of SINV replication by promoting cellular processes conducive to viral proliferation (45). A recent study by Ahlers et al. (32) using West Nile virus (WNV) infection in *Drosophila* S2 cells indicates a significant role for AKT phosphorylation during the infection process. The findings suggest that insulin-activated phosphorylation of AKT during WNV infection leads to the inhibition of RNAi and the activation of the JAK/STAT pathway to compensate for the loss of RNAi induction to restrict WNV replication (32). In *Drosophila* S2 cells, the exact role of AKT phosphorylation during DCV infection has not been explored. Since AKT plays an important role in immune response and inflammation, we hypothesized that AKT could be involved in host cellular responses to DCV infection. We observed increased AKT phosphorylation during DCV infection of naïve S2 cells, whereas the phosphorylation of AKT was reduced in the presence of Toll-9 (Fig. 4A). To address the effects of Toll-9 during DCV infection, we quantified AKT phosphorylation levels and intracellular DCV load. Western blot quantitation analysis revealed that the presence of Toll-9 significantly reduces AKT phosphorylation and intracellular DCV load during infection (Fig. 4B and C). To further validate that AKT phosphorylation regulates DCV infection, naïve S2 and Toll-9 OE cells were treated with AKT inhibitor VIII for 16 hours, followed by DCV infection. Pharmacological inhibition of AKT phosphorylation drastically reduced viral load from the DCV-infected S2 and Toll-9 OE cells (Fig. 4D). We also examined cells using fluorescence microscopy to show the effect of Toll-9 on intracellular viral load in both the presence and absence of AKT inhibitor. Our results suggest that in the absence of AKT inhibitor, CuSO_4_-induced Toll-9 OE cells had the lowest viral load compared to uninduced Toll-9 OE cells or naïve S2 cells (Fig. 4E). In the presence of AKT inhibitor, intracellular viral load was reduced in both cell types and treatment conditions (Fig. 4F). The observed reduction in viral load following treatment with the pharmacological inhibitor AKT VIII highlights that decreased AKT activation or phosphorylation significantly impairs viral replication. Next, to observe levels of infectious virus, cell culture supernatants were collected from DCV-infected naïve S2 cells and Toll-9 OE cells, both in the presence and absence of an AKT inhibitor. Our results show that the induction of Toll-9 expression or AKT inhibition resulted in the suppression of DCV replication (Fig. 4G). Taken together, these results suggest that Toll-9 may restrict DCV replication by modulating AKT dephosphorylation during infection. Alternatively, the observed reduction in AKT phosphorylation might be an indirect consequence of the decreased viral load.

### Toll-9 localizes in the endosomal vesicles

**Fig 5:**
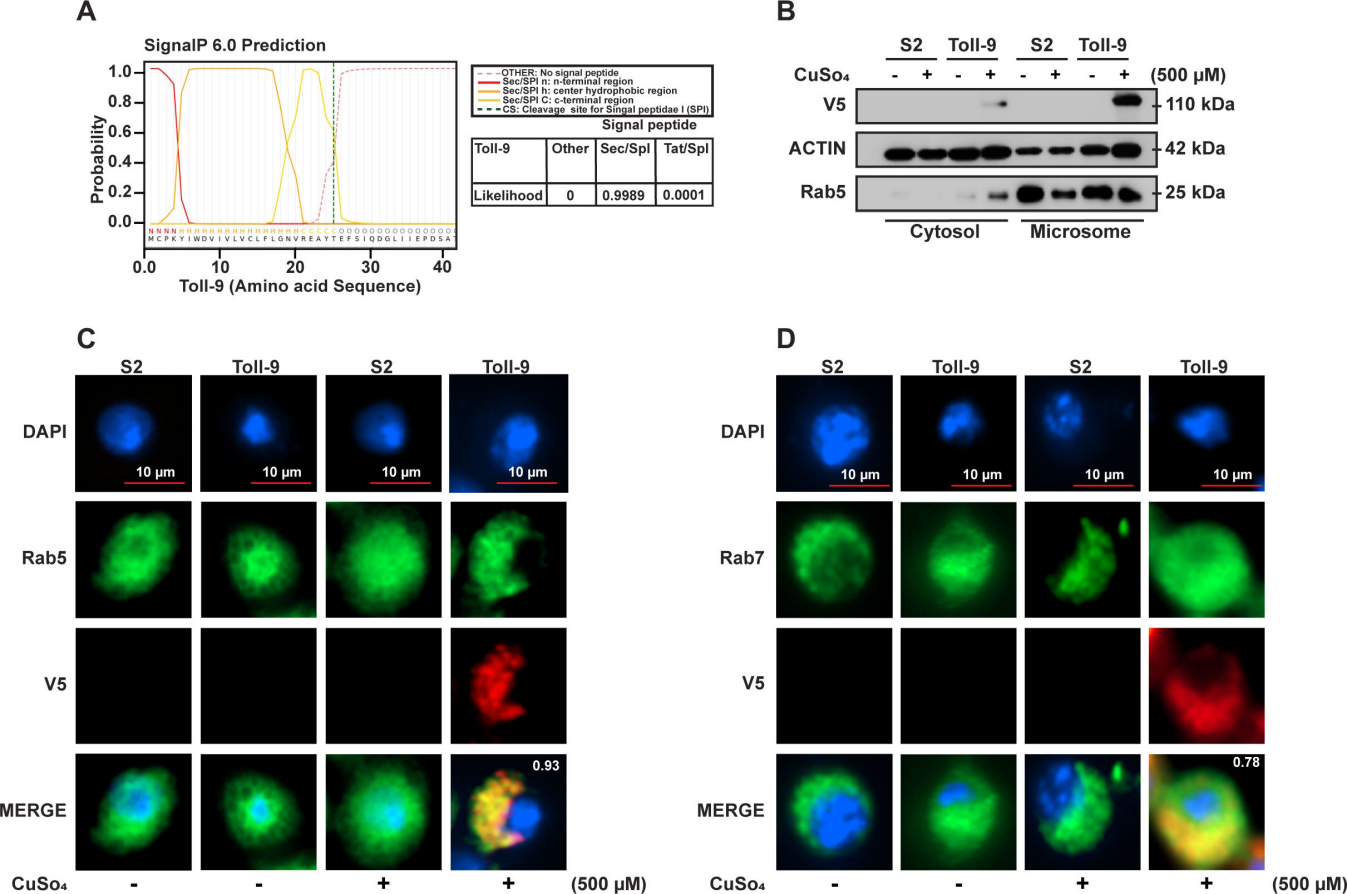
Subcellular localization of Toll-9. (**A**) *In silico* prediction of signal peptide in Toll-9 protein sequence. Red solid line indicates the predicted N-terminal region, orange solid line indicates the predicted center hydrophobic region, and yellow solid line indicates the predicted C-terminal region of signal peptide. Black dotted line indicates the cleavage site (CS) of the signal peptide. Sec/SPI: Sec translocon transported secretory signal peptide/Signal Peptidase I; Tat/SPI: Tat translocon transported Tat signal peptides/Signal Peptidase I. (**B**) Western blot analysis demonstrating the presence of Toll-9/V5 in endosomes. Endosomal fractions were identified using Rab5 as a microsomal marker, while Actin served as a cytosolic marker. (**C**) Micrographs showing colocalization of Rab5-early endosome marker (anti-Rab5, green) and Toll-9 (anti-V5 tag, red) in Toll-9 OE cells and S2 cells treated with or without CuSO_4_ (500 µM). DAPI (blue) is used to stain the nucleus of the cell. Pearson’s correlation coefficient for localization overlap is shown on the merge panel of the micrograph. (**D**) Micrographs showing colocalization of Rab7-late endosome marker (anti-Rab7, green) and Toll-9 (anti-V5 tag, red) in Toll-9 OE cells and S2 cells treated with or without CuSO_4_ (500 µM). DAPI (blue) is used to stain the nucleus of the cell. Pearson’s correlation coefficient for localization overlap is shown on the merge panel of the micrograph. The results are representative of three independent experiments.

Previous studies have shown that *Drosophila* Toll requires endocytosis for downstream signaling during both embryogenesis and innate immune responses (46, 47). Similarly, in humans, TLR4 activation by LPS triggers endocytic translocation, a process critical for both innate and adaptive immune signaling (48). Additionally, human TLR9 requires endocytic translocation for proteasomal processing by endosomal proteases, enabling downstream signal activation (49). Considering these previous studies, we first sought to explore the subcellular localization of Toll-9 in S2 cells. Toll-9 is a transmembrane protein, suggesting it may localize to intracellular membranes. We performed *in silico* analysis using the SignalP 6.0 database (50) and identified the presence of a signal peptide. Specifically, Toll-9 contains a signal peptide belonging to the Sec/SPI pathway that targets vesicular pathways with a high probability score of 0.9. The predicted signal peptide is located in the N-terminal region of Toll-9 and exhibits characteristic features such as an N-terminal sequence, a hydrophobic core, a C-terminal region, and a cleavage site for Signal Peptidase I (SPI) (Fig. 5A).

Next, we collected cellular fractions of naïve S2 and Toll-9 OE cells in the presence and absence of CuSO_4_. We performed western blot analysis on cytosolic and microsomal fractions of induced S2 and Toll-9 OE cells to detect the presence of Toll-9 protein in cytosolic or microsomal fractions. Our results show that Toll-9 is predominantly present in microsomal fractions, suggesting its localization either in the endoplasmic reticulum or a vesicular system such as endosomes (Fig. 5B). To visualize the intracellular localization of Toll-9, we performed fluorescence microscopy using Rab5 (early endosomal) and Rab7 (late endosomal) markers to localize Toll-9. Both early and late endosomal markers Rab5 and Rab7 colocalize with Toll-9, with Pearson’s correlation coefficients (PCC) of 0.93 and 0.78, respectively, suggesting the presence of Toll-9 in the endosomal compartment (Fig. 5C and D). Overall, our results indicate that Toll-9 localizes in endosomal compartments.

### Toll-9 interacts with dsRNA

**Fig 6:**
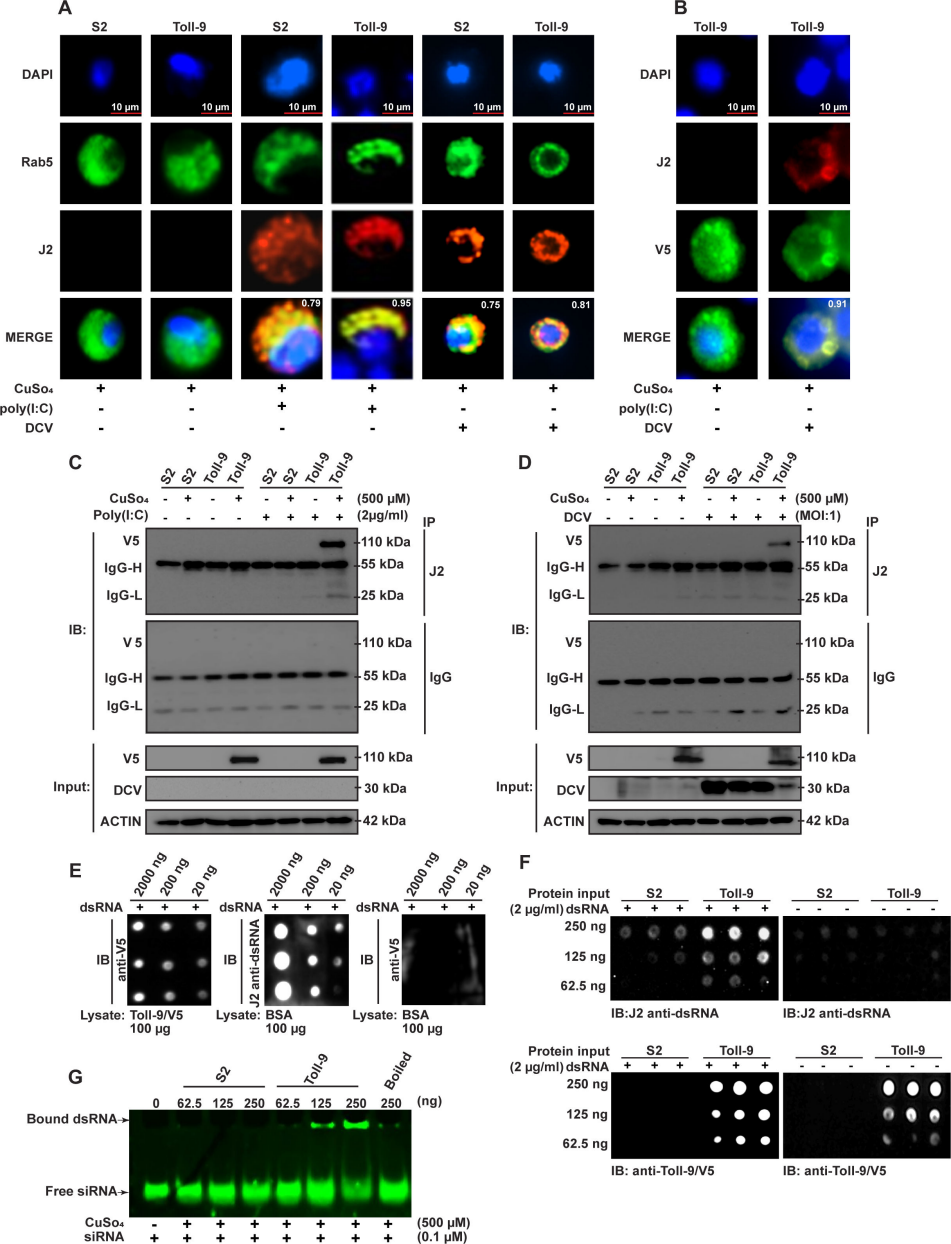
Toll-9 interacts with dsRNA in the endosomes. (**A**) Micrographs showing colocalization of Rab5 (anti-Rab5, green) and poly (I:C) or DCV dsRNA (J2 anti-dsRNA, red) in cells treated and untreated with CuSO_4_ (500 µM) in the presence and absence of poly (I:C) transfection or DCV infection. DAPI (blue) is used to stain the nucleus of the cell. Pearson’s correlation coefficient for localization overlap is shown on the merge panel of the micrograph. (**B**) Micrographs showing colocalization of DCV dsRNA (J2 anti-dsRNA, red) with Toll-9 (anti-V5 tag, green) in cells treated with CuSO_4_ (500 µM) in the presence and absence of DCV. DAPI (blue) is used to stain the nucleus of the cell. Pearson’s correlation coefficient for localization overlap is shown on the merge panel of the micrograph. (**C**) Western blot analysis using the indicated antibodies following immunoprecipitation of V5 tag (Toll-9) using anti-dsRNA (**J2**) antibody from naïve S2 cells and Toll-9 OE cells treated and untreated with poly (I:C) in the presence and absence of CuSO_4_ (500 µM). (**D**) Western blot analysis using the indicated antibodies following immunoprecipitation of V5 tag (Toll-9) using anti-dsRNA (**J2**) antibody of the lysate from uninfected and DCV-infected Toll-9 OE cells and S2 cells in the presence and absence of CuSO_4_ (500 µM). (**E**) dsRNA dot blot assay to detect dsRNA binding with purified Toll-9 protein. The dsRNA/Toll-9-V5 complex was blotted with an anti-V5 tag antibody. Control dsRNA-spotted blots were incubated with bovine serum albumin (BSA) and probed with J2 anti-dsRNA and anti-V5 tag antibodies. (**F**) Protein dot blot assay to detect purified Toll-9 protein in increasing concentration with dsRNA. Purified Toll-9 and S2 lysates were spotted onto membranes, incubated with or without dsRNA (2 µg/mL), and probed with J2 anti-dsRNA or anti-V5 antibodies. (**G**) RNA electrophoretic mobility shift assay (RNA EMSA) using purified Toll-9-V5 showing that Toll-9 binds to dsRNA in a concentration-dependent manner. The TBE gel represents the separation of bound and unbound dsRNA probes. The results are representative of three independent experiments.

RNA viruses produce dsRNA as a replication intermediate during their infection cycle (51–53). To investigate the subcellular localization of dsRNA, we treated S2 cells with the dsRNA analog poly (I:C) and performed immunofluorescence assays using the J2 dsRNA antibody and the endosomal marker Rab5. Our analysis revealed significant colocalization of dsRNA with endosomes in both naïve S2 cells and Toll-9 OE cells, as indicated by PCCs of 0.79 and 0.93, respectively (Fig. 6A, poly (I:C)-positive images). Interestingly, further analysis demonstrated that Toll-9 colocalized with dsRNA within endosomes in Toll-9 OE cells treated with poly (I:C), as suggested by a PCC of 0.95 (Fig. 6B, poly (I:C)-positive images). These findings support the hypothesis that Toll-9 may interact with dsRNA in endosomes.

During viral infection, replication intermediates such as dsRNA may also accumulate in the endocytic pathway of bystander, uninfected cells due to cell death or lysis. A previous report revealed that the viral dsRNA uptake by bystander cells can trigger systemic antiviral responses, potentially controlling viral replication (54). Moreover, cell-to-cell communication involving direct transfer of dsRNA and RNAi components through nanotube formations has been shown to mediate antiviral signal spread (55). To investigate the localization of viral dsRNA during DCV infection, we examined DCV-infected S2 cells and Toll-9 OE cells using the J2 dsRNA antibody and Rab5 as an endosomal marker. Immunofluorescence analysis indicated that viral dsRNA colocalized with Rab5-labeled endosomes, with PCC values of 0.75 in S2 cells and 0.81 in Toll-9 OE cells, respectively (Fig. 6A, DCV-positive images). This suggests that viral dsRNA is actively trafficked into endosomes during infection. Given that Toll-9 was detected in endosomes, we hypothesized that Toll-9 might directly interact with viral dsRNA. To test this, we performed immunofluorescence assays to examine the colocalization of Toll-9 with DCV-derived dsRNA in endosomes. The results revealed a strong colocalization between Toll-9 and viral dsRNA, as indicated by a PCC of 0.91 in DCV-infected Toll-9 OE cells (Fig. 6B, DCV-positive images). Collectively, these findings demonstrate that both poly (I:C) and viral dsRNA localize to endosomes and colocalize with Toll-9, suggesting that Toll-9 interacts with dsRNA within endosomes. This interaction may play a critical role in activating antiviral responses in *Drosophila* S2 cells during viral infection.

To examine the interaction between poly (I:C) or DCV-derived dsRNA with Toll-9, we performed immunoprecipitation assays using the J2 antibody. Lysates were prepared from naïve S2 cells and Toll-9 OE S2 cells, followed by treatment with poly (I:C), DCV infection, or their respective untreated controls, in the presence or absence of CuSO_4_. The J2 antibody successfully immunoprecipitated V5-tagged Toll-9 from lysates of poly (I:C)-treated or DCV-infected, CuSO_4_ induced S2 cells stably expressing Toll-9 (Fig. 6C and D). No IP signal was observed from naïve S2 cells or S2 cells stably expressing Toll-9 cells in the absence of poly (I:C) or DCV uninfected cells, suggesting that Toll-9 interacts with dsRNA.

To rule out the involvement of other interacting protein partners facilitating the binding of dsRNA with Toll-9, we affinity purified Toll-9 from the cell lysate of S2 cells stably expressing Toll-9 and used the identical purification condition for control lysate from naïve S2 cells in the presence of CuSO_4_ (Fig. S1). To confirm the direct binding of Toll-9 to dsRNA, we performed *in vitro* biochemical assays using purified V5-tagged Toll-9 from Toll-9 OE S2. We conducted a dot blot assay to validate direct Toll-9/dsRNA interaction. Increasing concentrations of dsRNA were immobilized on nitrocellulose membranes through UV crosslinking. The membranes were incubated with purified Toll-9 (1 µg/mL) or bovine serum albumin (BSA, 1 µg/mL) as a control. Immunoblotting with an anti-V5 antibody detected the binding of V5-tagged Toll-9 to dsRNA in a concentration-dependent manner, while no binding was observed with BSA (Fig. 6E).

Conversely, purified Toll-9 protein and S2 lysates, prepared under identical purification conditions, were spotted onto membranes at increasing concentrations. To assess dsRNA binding, nitrocellulose membranes were incubated with or without dsRNA (2 µg/mL) and probed with the J2 antibody to detect bound dsRNA. A second set of membranes was similarly treated and probed with an anti-V5 antibody to confirm the presence of V5-tagged Toll-9 spotted on the membrane. Only purified Toll-9 exhibited dsRNA binding in a concentration-dependent manner, with no binding observed in the S2 lysate (Fig. 6F).

To further confirm the direct interaction, we performed RNA electrophoretic mobility shift assays (EMSA) using a dsRNA probe and purified Toll-9. A concentration-dependent shift was observed with purified V5-tagged Toll-9, while no shift was detected with S2 cell lysates (Fig. 6G). In summary, our results demonstrate that Toll-9 binds dsRNA *in vitro* and *in vivo*, supporting its role as a dsRNA recognition receptor during viral infection.

## DISCUSSION

The structural similarity observed between Toll-9 and mammalian TLRs, alongside their recognized involvement in innate immune mechanisms, prompted the hypothesis that Toll-9 may play a role in innate immunity in *Drosophila* (18). However, Narbonne-Reveau et al. showed that the loss of Toll-9 does not affect basal AMP levels or their induction during bacterial infection (19). Nevertheless, recent studies highlight significant roles for Toll-9 in cell competition, aging, and neurodegeneration. Specifically, Toll-9 mediates apoptosis in unhealthy cells and promotes proliferation in fit cells (21, 22, 56). Despite these advances, Toll-9’s role in viral infections, its specific activator ligand, and the mechanistic details of its signaling cascade remain elusive and warrant further investigation.

DCV is one of the most studied natural pathogens of *Drosophila*, having coevolved with its host (57, 58). During infection, DCV activates various host immune pathways, including phagocytosis, RNAi, and JAK/STAT signaling (25, 58, 59). Given the structural similarities between Toll-9 and human TLRs, we hypothesized that it is involved in the antiviral response against DCV. Our study demonstrates Toll-9’s role in the antiviral response to the DCV infection in adult flies and S2 cells. A recent study using *Bombyx mori* Toll-9 (BmToll-9) reveals Toll-9 shares structural similarities with mammalian TLR4 and functions as a PRR that detects PAMP LPS (60). Previous research on *B. mori* immune-related genes has identified two Toll-9 variants with immune functions. Among these, BmToll-9-1 is more closely related to *Drosophila* Toll-9 and plays a crucial role in gut immune response (61). BmToll-9-1 expressing Bm5 cells, when exposed to dsRNA, exhibit significant upregulation of the key RNAi component gene *Dicer2* (62). RNAi is a crucial antiviral mechanism in arthropods (63), and it consists of two primary arms mediated by Dicer1 and Dicer2. Dicer1 plays a role in both RNA degradation and translational repression processes, whereas Dicer2 is primarily involved in RNA degradation. The loss of RNAi components *Dicer2, Argonaute2,* or *r2d2* increases susceptibility to viral infection (29). Our investigation revealed that DCV-infected transposable element insertion mutant *Toll-9^0024-G4^* mutant flies show reduced induction of *Dicer2* and *Argonaute2* correlated to higher mortality rates compared to infected isogenic parental control *w^1118^* flies. Contrary to expectations, our investigation revealed higher induction levels of JAK/STAT pathway components, including *Upd2, Upd3*, and *Vir1*, in mutant *Toll-9^0024-G4^* mutant flies compared to isogenic parental control *w^1118^* flies. This finding suggests that while the JAK/STAT pathway was upregulated, it alone was insufficient to provide adequate protection to the flies in the absence of induction of the RNAi pathway, as previously observed (33).

In this study, we observed a direct correlation between the presence of Toll-9 and the induction of *Dicer2* during DCV infection, indicating its potential role in mounting an antiviral response against DCV infection. Our observations indicate that during DCV infection of S2 cells, the presence of Toll-9 correlates with increased *Dicer2* expression and reduced *DCV replicase* ORF1 transcripts, suggesting an inhibitory role for Toll-9 in DCV replication. Although *Dicer2* is a key component of the RNAi pathway, our study doesn’t explore the specific effect of Toll-9 on RNAi activity during DCV infection. Previous studies suggest that Dicer2 can have an RNAi-independent process where it cleaves viral dsRNA and potentially confers an antiviral state (64). A recent study demonstrates that in shrimp infected with white spot syndrome virus, Dicer2 produces sequence-independent siRNAs from long dsRNA that serve merely as cleavage products without triggering RNAi (65). *Drosophila Dicer2* has also been shown to have RNAi-independent function and positively modulates Toll immune signaling against negative and positive strand RNA viruses (66). The report also suggests a novel link between Dicer2 and the Toll pathway during viral infection (66).

Many viruses promote host cell proliferation to prolong their replication and evade immune clearance, often through phosphorylation of AKT (39, 40, 67, 68). For example, in *Drosophila* S2 cells, Sindbis virus enhances AKT phosphorylation, which supports virus replication (45). Insulin-mediated PI3K/AKT signaling also regulates the expression of RNAi genes *Dicer2* and *Argonaute2* via FoxO during West Nile and Zika virus infection (32, 69). Our study demonstrates that the presence of Toll-9 during DCV infection of S2 cells has inhibitory effects on AKT phosphorylation and viral load. Notably, to our knowledge, this is the first study to examine AKT’s role in DCV replication in *Drosophila* S2 cells. We observed that infection of S2 cells expressing Toll-9 with DCV results in the induction of *Dicer2* and the inhibition of AKT phosphorylation, correlated to reduced DCV replication. The heightened induction of *Dicer2* in the presence of Toll-9 during DCV infection could be attributed to the presence of dsRNA intermediates, which are known to induce *Dicer2* expression (62). Furthermore, it can be hypothesized that the sharp increase in *Dicer2* expression is linked to reduced AKT phosphorylation, a key regulator of FoxO translocation from the nucleus to the cytosol. The reduction in AKT phosphorylation lifts its inhibitory effect on FoxO nuclear translocation and retention, allowing FoxO to remain in the nucleus and enhance *Dicer2* expression (70). Additionally, the study shows that during RNA virus infection, ectopic expression of Dicer2 in dFoxO mutant flies restores the antiviral response observed in wild-type dFoxO, further supporting its role in *Dicer2* regulation (70). A recent study on *Drosophila* dopaminergic neuronal cell survival shows that overexpression of Toll-1 and Toll-7 induces autophagy by dephosphorylating AKT via activation of Protein Phosphatase 2A, suggesting the role of Toll signaling in regulating phosphorylation of AKT (71). Previous studies from our lab also demonstrate that the inhibition of AKT phosphorylation using AKT inhibitor decreases phosphorylation of FoxO, causing nuclear retention and induction of *Dicer2* and *Ago2* (32, 69). Furthermore, pharmacological inhibition of AKT during DCV infection in S2 cells mimics the restrictive effect of Toll-9, underscoring Toll-9’s role in inhibiting DCV infection through AKT signaling. However, our data suggest an association between Toll-9 expression and reduced AKT phosphorylation and DCV loads. Alternatively, the reduction in AKT phosphorylation may be a downstream effect rather than a direct consequence of Toll-9 expression. It could also result from decreased DCV replication in the presence of Toll-9. Mammalian TLRs exhibit diverse subcellular localization, with TLRs 1, 2, 4, 5, and 6 being primarily localized on the cell membrane interacting with extracellular PAMPs, while TLRs 3, 7, 8, 9, and 10 are predominantly found in endosomes or lysosomes where they sense intracellular PAMPs such as nucleic acids (28, 72). Our findings indicate that *Drosophila* Toll-9 localizes to both early and late endosomes, suggesting a role in nucleic acid sensing. This hypothesis is further supported by Toll-9’s ability to bind to poly (I:C), a double-stranded RNA analog and pathogenic dsRNA derived from DCV, implying that it can detect viral dsRNA, similar to human TLR10 (28). Since this study did not utilize recombinant Toll-9 expressed in a heterologous system, such as a bacterial expression system, the possibility remains that an additional protein copurified with Toll-9 may facilitate the interaction between Toll-9 and dsRNA. Although the likelihood of another protein playing a role in this interaction appears to be very low, it cannot be entirely ruled out. During DCV infection, the detection of dsRNA by Toll-9 in endosomes triggers signaling that restricts viral replication. This signaling leads to the upregulation of *Dicer2,* causing a reduction of viral loads. This parallels the mechanism in mammalian systems where TLRs mediate immune responses, underscoring the evolutionary significance of Toll-9 in antiviral defenses.

In summary, we demonstrated that Toll-9 is crucial for antiviral defense against DCV infection in S2 cells. Toll-9 restricts DCV replication by inducing expression of the RNAi pathway gene *Dicer2* and restriction of AKT phosphorylation, leading to reduced intracellular viral loads. Our data demonstrate that Toll-9 is localized in the endosomes, similar to human TLR10, and it can bind to dsRNA. This investigation will shed light on Toll-9’s potential role in modulating cellular immune responses against viral infections, providing valuable insights into its function in the innate immune system of *Drosophila*.

### Limitations of the study

In this study, we identified that dsRNA can be detected by Toll-9, which upon binding, induces *Dicer2* expression and influences AKT phosphorylation, imparting an antiviral state against DCV infection of S2 cells. In this study, Toll-9 protein was purified from S2 cells rather than a heterologous system for biochemical studies. While this leaves the possibility that an endogenous protein may have co-purified with Toll-9 and contributed to its interaction with dsRNA, the likelihood of such involvement is minimal. Therefore, how the signal proceeds downstream after dsRNA binding and how the components of the Toll-9 signaling pathway are involved in AKT modulation remain to be explored. The observed AKT dephosphorylation in the presence of Toll-9 could result from direct Toll-9 signaling activation or indirectly from the reduction in DCV load. Further experiments are necessary to fully elucidate this mechanistic link. The regulation of AKT phosphorylation is tightly controlled by various kinases and phosphatases involved in the PI3K/AKT pathway. Therefore, the results from this study can be expanded in future research efforts to examine the functional link between Toll-9 mediated RNAi induction and the molecular mechanism of Toll-9 signal transduction for AKT regulation during viral infection.

## MATERIALS AND METHODS

### Fly stocks and insect cell culture

*Drosophila melanogaster* genotypes used were *w^1118^* and *w^1118^; PBac{w^+mC^ = IT.GAL4} Toll-9^0024-G4^* (Bloomington *Drosophila* Stock Center 5905 and 62581). Flies were grown in standard meal agar fly food and maintained at 23°C and 68% humidity under a 12 hour/12 hour light/dark cycle. *Drosophila* hemocyte-derived S2 cells were maintained at 28°C in tissue culture flasks containing Schneider’s *Drosophila* medium (Gibco, Waltham, MA) supplemented with 10% heat-inactivated fetal bovine serum (FBS) (HyClone), 100 U/mL of penicillin, 100 µg/mL of streptomycin, and 0.25 µg/mL of amphotericin B (Fungizone) antimycotic (Life Technologies, Waltham, MA).

### DCV infection of *Drosophila melanogaster*

Age-matched 4- to 7-day-old flies were injected with DCV (50,000 TCID_50_) or mock (Saline), and mortality was evaluated for a period of 30 days. For injections, flies were anesthetized with CO_2_ and injected with 23 nL of virus or phosphate-buffered saline (PBS) using a pulled 0.53 mm glass needle and an automatic nanoliter injector (Drummond Scientific, Broomall, PA). Individual flies were injected at the ventrolateral surface of the fly thorax and placed into new vials. After the injections, the adult flies were monitored daily for mortality and collected at different times post-infection to assess viral load or host responses. Survival curves were performed using a minimum of 80 flies per condition, including at least three experimental replicates. The viral titer was analyzed observing the cytopathic effect of serially diluted cell culture supernatant from DCV-infected S2 cells by calculating TCID_50_/mL.

### DCV infection of *Drosophila* S2 cells

To prepare S2 cells for DCV infection, cells were seeded at the required density in 1× complete cell culture medium (without antibiotics or antifungal agents) and incubated for 16 hours or overnight. Before infection, the culture medium was carefully removed, and the cells were washed twice with incomplete cell culture medium (1× S2 cell culture medium without FBS) to remove residual serum components. For infection, S2 cells were incubated with infection medium (1× S2 cell culture medium without FBS, containing DCV at a multiplicity of infection of 1) for 1 hour. During this period, the culture flask was gently inverted every 15–20 minutes to ensure uniform exposure of cells to the virus. Following the incubation, the infection medium was removed, and the cells were washed three times with incomplete cell culture medium to remove unbound viruses. Finally, the medium was replaced with fresh complete cell culture medium, and the cells were incubated under standard culture conditions for subsequent experiments.

### Gene expression analysis

The relative expression of each RNAi and JAK/STAT pathway genes was determined in adult flies by qRT-PCR. Six biological replicates of S2 cells or 3–5 flies per each condition were lysed in Trizol Reagent (ThermoFisher 15596), RNA was isolated by column purification (ZymoResearch R2050), and cDNA was synthesized using the iScript Reverse Transcriptase kit (Bio-Rad, Hercules, CA). qRT-PCR was performed using SYBR Green PCR master mix (Bio-Rad). For gene expression analysis using qRT-PCR, the 2^-ΔΔCt^ method was used. For all gene expression data, *Rpl32* was used as an endogenous control. In the case of fly infection experiments, *w^1118^* mock infection was used for normalization with target genes at each time point.

*RpL32* (5′-GACGCTTCAAGGGACAGTATCTG-3′ and 5′-AAACGCGGTTCTGCATGAG-3′) *Vir1* (5′-GATCCCAATTTTCCCATCAA-3′ and 5′-GATTACAGCTGGGTGCACAA-3′), DCV (5′-TCATCGGTATGCACATTGCT-3′ and 5′-CGCATAACCATGCTCTTCTG-3′), *Ago2* (5′-CCGGAAGTGACTGTGACAGATCG-3′ and 5′-CCTCCACGCACTGCATTGCTCG-3′), *Upd2* (5′-CCTATCCGAACAGCAATGGT-3′ and 5′-CTGGCGTGTGAAAGTTGAGA-3′), *Upd3* (5’-GCCCTCTTCACCAAACTGAA-3′ and 5′-TCGCCTTGCACAGACTCTTA-3′), *Dcr2* (5’-GTATGGCGATAGTGTGACTGCGAC-3′ and 5′-GCAGCTTGTTCCGCAGCAATATAGC-3′), *Toll-9* (5′-CTTTGAGGTCAGCAAGGAGC-3′ and 5′-ACACTGATCTCTGGAGTTGA-3′).

### Plasmid constructs and transfection into S2 cells

The coding regions of *Drosophila Toll-9* (*CG5528*) were amplified from *Drosophila* Genomics Resource Center clone IP19811. Amplified PCR fragments were inserted into the metallothionein promoter-driven pMT-V5-His vector (Invitrogen). The generated vector was co-transfected with a pCoBlast blasticidin resistance vector with a ratio of 19:1, and cells were selected in a blasticidin-containing medium according to the manufacturer’s instructions. After establishing several stably transfected lines, the expression level was checked with CuSO_4_ induction (500 µM), and the cells were used for the experiments performed in this study.

### Immunoblotting

Flies or S2 cells were homogenized in RIPA buffer (25 mM Tris-HCl [pH 7.6], 150 mM NaCl, 1 mM EDTA, 1% NP-40, 1% sodium deoxycholate, 0.1% SDS, 1 mM Na_3_VO_4_, 1 mM NaF, 0.1 mM phenylmethylsulfonyl fluoride, 10 µM aprotinin, 5 µg/mL leupeptin, 1 µg/mL pepstatin A). Total protein was determined using the bicinchoninic acid assay (Pierce, Waltham, MA). Equal amounts of protein were subjected to SDS-PAGE. The proteins were transferred to a polyvinylidene difluoride membrane and blocked in 5% BSA in 0.1% Tween 20–Tris-buffered saline. The membrane was incubated with antibodies against DCV capsid (1:1,000; Abcam ab92954), V5 tag (1:5,000; ThermoFisher Scientific R960-25), actin (1:5,000; Sigma A2066), and rab5 (1:1,000; Abcam ab31261) antibodies overnight at 4°C. Antibody-bound proteins were detected using anti-rabbit secondary antibodies conjugated to horseradish peroxidase. The blots were developed by chemiluminescence using a luminol enhancer solution (ThermoFisher).

### Fluorescence microscopy

The infected cells were fixed for 20 minutes in 4% formaldehyde, followed by permeabilization for 20 minutes in 0.1% Triton X-100. The cells were blocked in PBS containing 10% FBS and incubated with antibodies against Rab5 (1:50; Abcam ab31261), Rab7 (1:20; Developmental Studies Hybridoma Bank Rab7), DCV Capsid (1:200; Abcam ab92954), dsRNA J2 (1:200; Jena Biosciences RNT-SCI-10010200), and V5 tag (1:200; ThermoFisher Scientific R960-25) for overnight at 4°C. The cells were washed and incubated with rabbit or mice Alexa Fluor-488 (ThermoFisher Scientific A11034 or A11035)- and rabbit or mice Alexa Fluor-594 (ThermoFisher Scientific A11029 or A11030)-conjugated secondary antibodies for 1 hour at room temperature. The cells were washed, incubated with DAPI (4′,6-diamidino-2-phenylindole) (Sigma D9542) for 15 minutes, and mounted onto microscope slides. Images were obtained using the Biotek Cytation 3 fluorescent microscope reader and analyzed using Image J (73). A 20× objective was used for capturing images.

### Poly (I:C) delivery

To deliver poly (I:C) to the S2 cells or Toll-9 OE cells, 10^6^ cells were incubated with 5 µg/mL poly (I:C) as described previously (74). Cell culture media was removed before the treatment of poly (I:C) and replaced with the incomplete cell culture media without FBS to starve the cells for 1 hour to increase the delivery of poly (I:C). Incomplete cell culture media was replaced with complete cell culture media, and cells were allowed to grow for 48 hours, followed by induction with 500 µM CuSO_4_ for 24 hours. The cells were harvested and lysed using RIPA buffer with protease inhibitors.

### Immunoprecipitation

For poly (I:C) and interacting protein complex immunoprecipitation assay, 200 µg lysate was incubated with 2 µg anti-dsRNA J2 antibody for 16 hours at 4°C on a rotating nutator. After incubating the cell lysate and anti-dsRNA J2 antibody, 40 µL of 50% vol/vol protein A agarose beads were added to the mixture and incubated for 3 hours at 4°C on a rotating nutator. Bead-antibody-protein complexes were washed three times in 1× RIPA buffer and then incubated for 10 minutes at 97°C with 2× loading dye. Lysates were centrifuged for 5 minutes at 13,000 × *g* to remove the beads and subjected to western blotting analyses. Similarly, for DCV dsRNA and its interacting protein complex immunoprecipitation assay, 400 µg lysate was used, and the identical protocol mentioned for poly (I:C) was utilized for the assay.

### Organelle isolation

To isolate cytosol and microsome, cells were washed twice with PBS at 300 g for 5 minutes at 4°C. After washing once with PBS, cells were washed with 1 mL 0.9% sodium chloride solution at 300 × *g* for 5 minutes at 4°C. The pellet was resuspended in the lysis buffer provided in the kit, and cells were fractionated using the Qproteome-mitochondria isolation kit according to manufacturer’s instructions (Qiagen 37612).

### AKT inhibitor VIII

We utilized AKT inhibitor VIII (MedChemExpress; HY-10355), a cell-permeable, reversible, and highly selective inhibitor of AKT1, AKT2, and AKT3 activity, to inhibit AKT signaling in *Drosophila* S2 cells. For experiments requiring AKT inhibition, S2 cells were pre-treated with 10 µM AKT inhibitor VIII for 16 hours. Following this, the cells were washed twice with incomplete culture medium (1× S2 cell culture medium without FBS) to remove residual inhibitors before DCV infection. After the infection, the unbound DCV was removed by washing the cells twice with an incomplete culture medium. A fresh complete culture medium containing 10 µM AKT inhibitor VIII was then added to the cells and maintained for the duration of the experimental time points.

### Dot blot dsRNA-protein binding assay

To assess the binding of dsRNA to Toll-9, increasing concentrations of dsRNA (2,000, 200, and 20 ng) were spotted onto a positively charged nitrocellulose membrane (Amersham Hybond-N+). The membrane was air-dried for 20–30 minutes and then UV-crosslinked at 120,000 μJ/cm² for 25–30 seconds to immobilize the dsRNA. Following crosslinking, the membrane was blocked with 1× dsRNA binding (Tris 50 mM, NaCl 150 mM, MgCl_2_ 1 mM, 1 mM DTT) with 2.5% BSA for 1 hour at room temperature. After blocking, the membrane was washed gently with the same buffer for 5 minutes to remove unbound protein. The membrane was then incubated overnight at 4°C on a rotating nutator with 100 µg of Toll-9 purified lysate fraction or 100 µg of BSA (both in 50 mM Tris, 150 mM NaCl, 50 mM Imidazole, pH 7.0) as a control. The next day, the membrane was washed three times with 1× dsRNA binding buffer to remove unbound protein and incubated with primary antibodies for 3 hours at room temperature: anti-V5 mouse (1:5,000) antibody for Toll-9 binding and J2 anti-dsRNA mouse (1:5,000) and anti-V5 mouse (1:5,000) antibody for BSA control blots. The membrane was washed three times with 1× PBST (PBS + 0.1% Tween-20) for 5 minutes each and then incubated with an HRP-conjugated anti-mouse IgG secondary antibody (1:10,000) for 1 hour. After washing five times with 1× PBST (5 minutes each), the membrane was developed using a chemiluminescence detection kit with a luminol enhancer solution (ThermoFisher).

### Dot blot protein-dsRNA binding assay

For the reciprocal protein-dsRNA binding assay, purified fractions of Toll-9 and S2 cell lysate (50 mM Tris, 150 mM NaCl, 50 mM Imidazole, pH 7.0) were spotted onto four identical nitrocellulose membranes (0.45 µm; ThermoFisher). The membranes were air-dried for 20–30 minutes, blocked with 5% skim milk in 1× PBS for 30 minutes at room temperature, and then washed three times with 1× PBS for 5 minutes each. The membranes were equilibrated in 1× dsRNA binding buffer for 15 minutes and rinsed three times with the same buffer. The membranes were then incubated with or without 2 µg/mL dsRNA diluted in 1× dsRNA binding buffer for 30 minutes at room temperature with gentle shaking. After incubation, the membranes were washed three times with 1× dsRNA binding buffer (5 minutes each) to remove unbound dsRNA. For detection, the membrane was incubated overnight at 4°C with the primary antibodies J2 anti-dsRNA antibody (1:5,000) to detect dsRNA binding to Toll-9 and anti-V5 antibody (1:5,000) to confirm the presence of Toll-9 protein in the presence and absence of dsRNA. The next day, the membranes were washed three times with 1× PBST (5 minutes each), incubated with an HRP-conjugated anti-mouse IgG secondary antibody (1:10,000) for 1 hour, and washed again five times with 1× PBST (5 minutes each). Chemiluminescence detection was performed using a luminol enhancer solution (ThermoFisher).

The dsRNA details are as follows: length, 267 bp; sequence, 5′GUGCACGUAUAACAACAGAUCGAUAUACCUGAUUGUGGGAUUCCUUCUCGUAGCUUUUUUUCGAAUUUCGGUUACCGGGAAUUACAGGAACGUAAUGCCCACGACGCUGUUUCUGUUCCAAAUGCCGCUCUACUGGAUAUGGAGCUUUACCGAUAUGGACCAAAGCACCCUGAGCUACUCGCACUGGAUACGUGACUCCCAUGGACUAGACUAUGCGGCCGGAAUGGCCUCCAACUACUUUCACGGCUACCUCAAACUUUCACUGCC-3′.

### RNA electrophoretic mobility shift assay

The ability of Toll-9 to bind with dsRNA was demonstrated utilizing separation of bound and unbound dsRNA probe on non-denaturing TBE (Tris-Borate Edta) gel electrophoresis. For RNA EMSA assay, identical purified fraction (Tris 50 mM, NaCl 150 mM, and 50 mM Imidazole; pH 7.0) from Toll-9 and S2 cell lysate was incubated with ~21–24 bp dsRNA in 1× dsRNA binding buffer for 15 minutes at room temperature. The reaction mixture was then loaded onto the 12% TBE gel, and electrophoresis was performed in 1× TBE buffer at 90 V for 25 minutes. The electrophoresed gel was then incubated with 3× gel green stain in nuclease-free water (Biotium) for 30 minutes with gentle shaking for staining. The green-stained gel was then rinsed twice with nuclease-free water and imaged on the gel documentation system (Analytikjena).

### Quantification and statistical analysis

An unpaired two-tailed Student’s t test assuming unequal variance was utilized to compare means of quantitative data. Mortality curves were analyzed by the log-rank (Mantel-Cox) test using GraphPad Prism (GraphPad Software, Inc.). Analysis and graph generation were done using GraphPad Prism 9. The statistical method used was one-way analysis of variance (ANOVA) with the Holm-Sidak test for multiple comparisons, unless otherwise indicated. Plotted is mean intensity ± SEM. Levels of significance are depicted by asterisks in the figures: ^∗^*P* < 0.05; ^∗∗^*P* < 0.01; ^∗∗∗^*P* < 0.001; ^∗∗∗∗^*P* < 0.0001. At least three or more biological replicates were analyzed for each experimental condition unless otherwise indicated. For Pearson’s colocalization coefficient, five micrographs were analyzed using ImageJ (75).

## Data Availability

This study did not generate new nucleotide sequence, microarray, proteomic data, or RNA sequencing data. All relevant data supporting the findings of this study are available within the article and its supplemental material. Additional details can be obtained from the corresponding author upon reasonable request.

## SUPPLEMENTAL MATERIAL

Figure S1 jvi.02214-24-s0001.pdf Toll-9-V5/His affinity chromatography purification.

ASM does not own the copyrights to Supplemental Material that may be linked to, or accessed through, an article. The authors have granted ASM a non-exclusive, world-wide license to publish the Supplemental Material files. Please contact the corresponding author directly for reuse.
