# Supplementary material for: Drosophila melanogaster Toll-9 elicits antiviral immunity against Drosophila C virus
Source: J Virol. 2025 May 14;99(6):e02214-24. doi: 10.1128/jvi.02214-24 (PMC12172494; doi:10.1128/jvi.02214-24)
Supplement: Figure S1 — Toll-9-V5/His affinity chromatography purification. [file jvi.02214-24-s0001.pdf]

Supplementary Figures

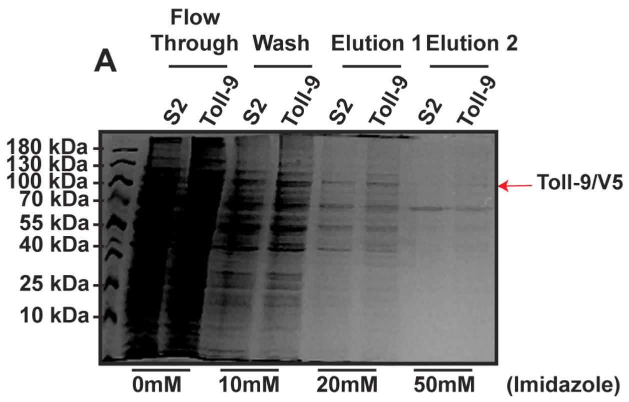

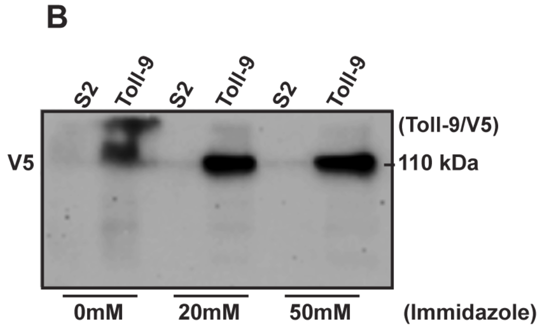

Supplementary Figure S1. Toll-9-V5/His affinity chromatography purification.

(A) SDS page for Ni-NTA based affinity chromatography purified Toll-9-V5/His from $CuSO_4$ (500 μM) treated Toll-9 OE and S2 cells lysate.

(B) Western blot shows the presence of purified Toll-9 using V5 antibody in indicated eluted fractions using wash buffer containing mentioned imidazole concentrations.

Data are representative from three independent experiments.

Supplementary Method

Purification of His-Tagged Toll-9 Protein Using Ni-NTA Affinity Chromatography

S2 cells expressing His-tagged Toll-9 protein and naïve S2 cells (control) were harvested by centrifugation at 4°C, 5000 x g for 10 minutes. The resulting cell pellets were resuspended in lysis buffer containing 50 mM Tris (pH 7.0), 150 mM NaCl, and 0.1% Triton X-100, supplemented with a 1X protease inhibitor cocktail. The cells were lysed using a Dounce homogenizer with 20 strokes to ensure efficient disruption. The lysate was centrifuged at 4°C, 12,000 x g for 30 minutes to remove cellular debris, and the supernatant from both the His-tagged Toll-9 protein-expressing cells and naïve S2 cells was collected.

The clarified supernatant was applied to Ni-NTA agarose resin equilibrated with lysis buffer. The resin-supernatant mixtures were incubated with gentle mixing at 4°C overnight to facilitate binding of the His-tagged protein to the resin. The following day, the resin-lysate mixtures were transferred to centrifuge columns and washed with 10–15 column volumes of wash buffer containing 50 mM Tris (pH 7.0), 150 mM NaCl, and 10 mM imidazole to remove non-specifically bound proteins.

The His-tagged Toll-9 protein and control lysates from naïve S2 cells were eluted by applying elution buffer containing 50 mM Tris (pH 7.0) and 150 mM NaCl, with varying concentrations of imidazole ranging from 10 to 50 mM.
